# Revitalizing quality of life: a case report on the beneficial impact of comprehensive rehabilitation therapy in treating upper-limb lymphedema following breast cancer surgery

**DOI:** 10.3389/fonc.2023.1046003

**Published:** 2023-06-16

**Authors:** Yan-Fang Sui, Lang-Qian Tong, Xia-Fei Lin, Hai-Xia Wu, Jing-Qin Shi, Shan-Shan Wang, Bu-He Tang, Zhen-Hua Song

**Affiliations:** ^1^ Department of Rehabilitation Medicine, Affiliated Haikou Hospital of Xiangya Medical College, Central South University, Haikou, China; ^2^ Department of Nuclear Medicine, Affiliated Haikou Hospital of Xiangya Medical College, Central South University, Haikou, China; ^3^ Department of Rehabilitation Medicine, Hainan Sino-german orthopaedic Hospital, Haikou, China

**Keywords:** breast cancer, comprehensive rehabilitation, functional brace, lymphedema, seven-step decongestion therapy

## Abstract

**Objective:**

To underscore the paramount significance of incorporating comprehensive rehabilitation therapy as a crucial aspect of managing lymphedema caused by breast cancer surgery, and to illuminate our first-hand experience and insights gained in utilizing this approach.

**Methods:**

We present a case report of a breast cancer survivor who had been suffering from persistent left upper-limb edema for over 15 years, who was effectively treated with a combination of conventional rehabilitation (seven-step decongestion therapy) and a comprehensive rehabilitation program (seven-step decongestion therapy, along with core and respiratory function training, as well as functional brace wearing). The efficacy of the rehabilitation therapy was evaluated through a comprehensive assessment

**Results:**

Although the patient underwent the conventional rehabilitation program for one month, only limited improvement was observed. However, after an additional month of comprehensive rehabilitation treatment, the patient exhibited significant improvement in both lymphedema and the overall function of the left upper limb. The patient’s progress was quantified by measuring the reduction in arm circumference, which demonstrated a notable decrease. Furthermore, improvements in joint range of motion were observed, with forward flexion of the shoulder enhancing by 10°, forward flexion improving by 15°, and elbow flexion increasing by 10°. In addition, manual muscular strength tests revealed an increase in strength from Grade 4 to Grade 5. The patient’s quality of life was also significantly improved, as evidenced by the increase in the Activities of Daily Living score from 95 to 100 points, the increase in the the Functional Assessment of Cancer Therapy: Breast score from 53 to 79 points, and the decrease in the Kessler Psychological Distress Scale score from 24 to 17 points.

**Conclusion:**

While seven-step decongestion therapy has been shown to be effective in reducing upper-limb lymphedema caused by breast cancer surgery, it has limitations in treating more chronic cases of the condition. However, when combined with core and respiratory function training and functional brace wearing, seven-step decongestion therapy has been shown to be even more effective in reducing lymphedema and improving limb function, ultimately leading to significant improvements in quality of life.

## Introduction

Breast cancer is the most frequently diagnosed cancer in women worldwide and continues to be a major global health concern (1). Breast cancer incidence rates have been rapidly increasing in China, making it one of the countries with the fastest-growing rates of breast cancer globally (2–4). The primary components of breast cancer surgery typically involve lumpectomy and/or axillary lymph node dissection (2). Lymphedema can result from a variety of causes including surgery, chemotherapy, radiotherapy, trauma, infection, obesity, and other personal factors (4, 5).

Breast cancer-related lymphedema (BCRL) affects about 25% of women following breast cancer surgery (1, 6). This condition can have a significant impact on a patient’s quality of life and physical function (5–7).The typical treatments for breast cancer-related lymphedema (BCRL) involve lymphatic drainage, elastic bandaging, exercise therapy, and other rehabilitation therapies. However, there is limited research on comprehensive rehabilitation therapy for this condition (8–13).Therefore, a case is presented to underscore the significance of comprehensive rehabilitation and share the practical knowledge gained from it. The presented case involves a patient who experienced left upper-limb swelling for over 15 years following breast cancer surgery. Although conventional therapy yielded limited improvements, the patient’s lymphedema and function markedly improved after undergoing one month of comprehensive rehabilitation.

## Case presentation

A 51-year-old female patient who had undergone modified radical resection of her left breast and had been experiencing left upper limb swelling and limited mobility for 15 years was admitted to the Affiliated Haikou Hospital of Xiangya Medical College. The patient underwent modified radical resection of the left breast on April 19, 2006, following preoperative chemotherapy, due to a mass found on the left breast. The specific details regarding the intraoperative lymph node dissection are unknown. However, intraoperative pathology confirmed the mass as Grade 3 invasive ductal cancer, and the patient received postoperative concurrent chemotherapy and radiation. The patient experienced redness, swelling, pain, and increased skin temperature in the left upper limb six months after the surgery. This was diagnosed as cellulitis of the left upper limb caused by *Staphylococcus aureus* infection. The patient received appropriate treatments that provided some relief from the pain, but these treatments were irregular. Nonetheless, the left upper limb remained swollen. As the patient was left-handed, the swelling of her left hand had a serious impact on the quality of life. Consequently, she sought rehabilitation treatment at our hospital.

**Figure 1:**
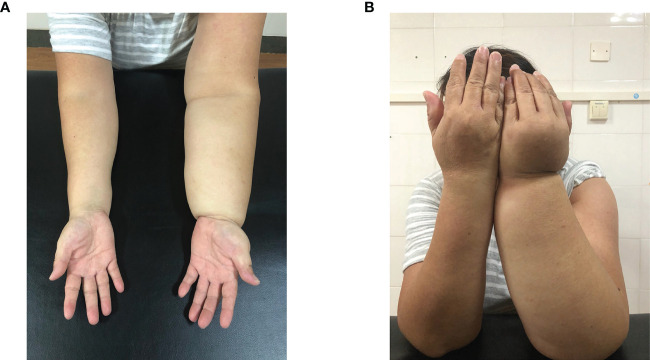
Left Upper Limb Lymphedema. (**A**: Palmar Side of Forearm, **B**: Dorsal Side of Forearm).

**Figure 2:**
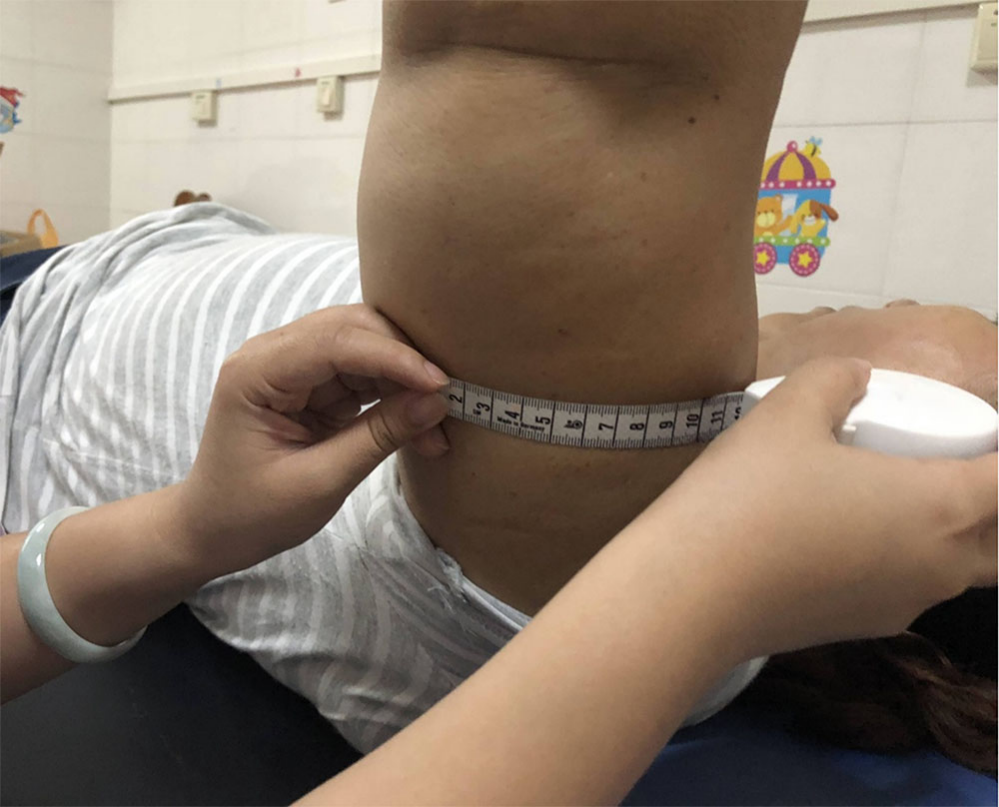
Measurement Method for Left Upper Limb Lymphedema.

**Figure 3:**
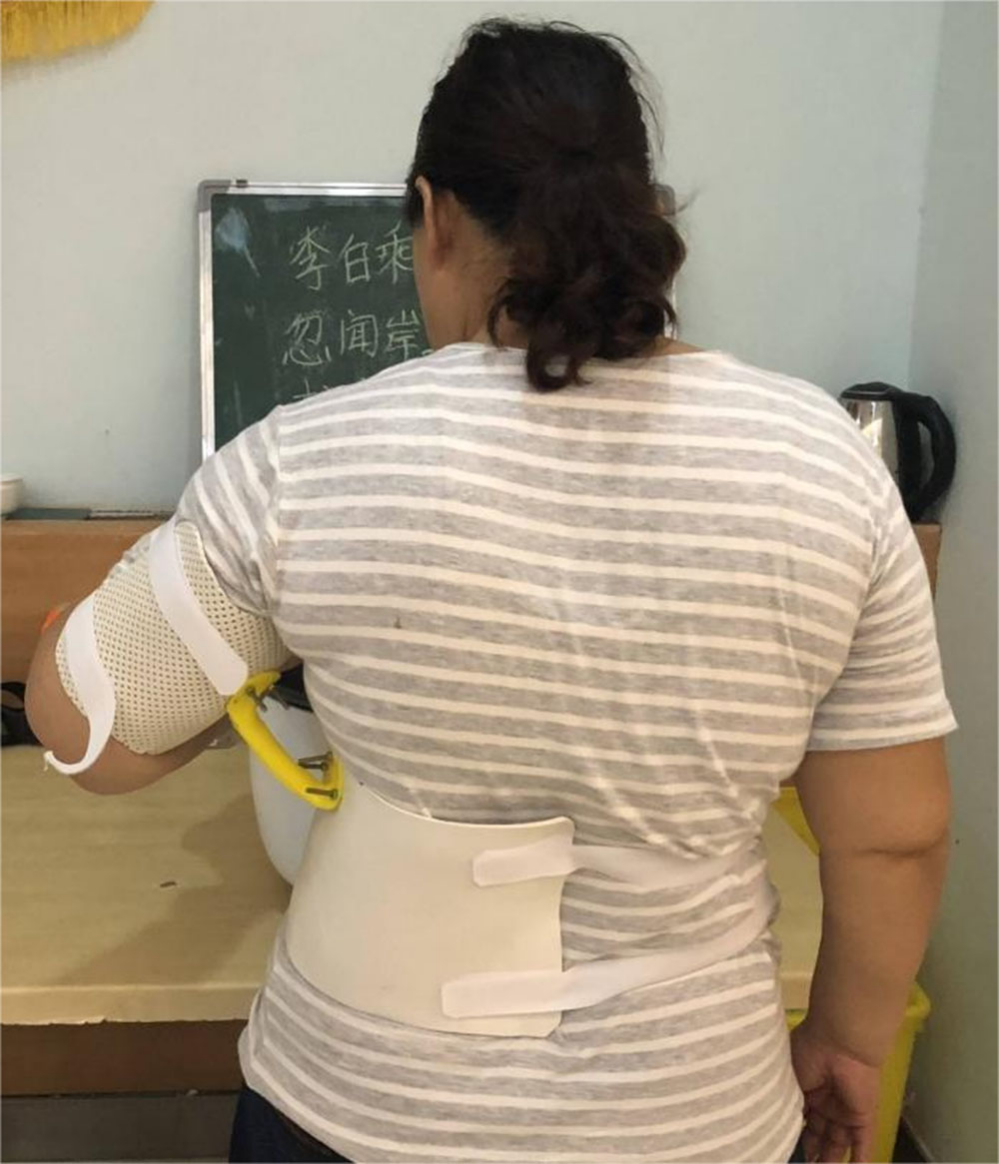
Functional Brace Wearing.

**Figure 4:**
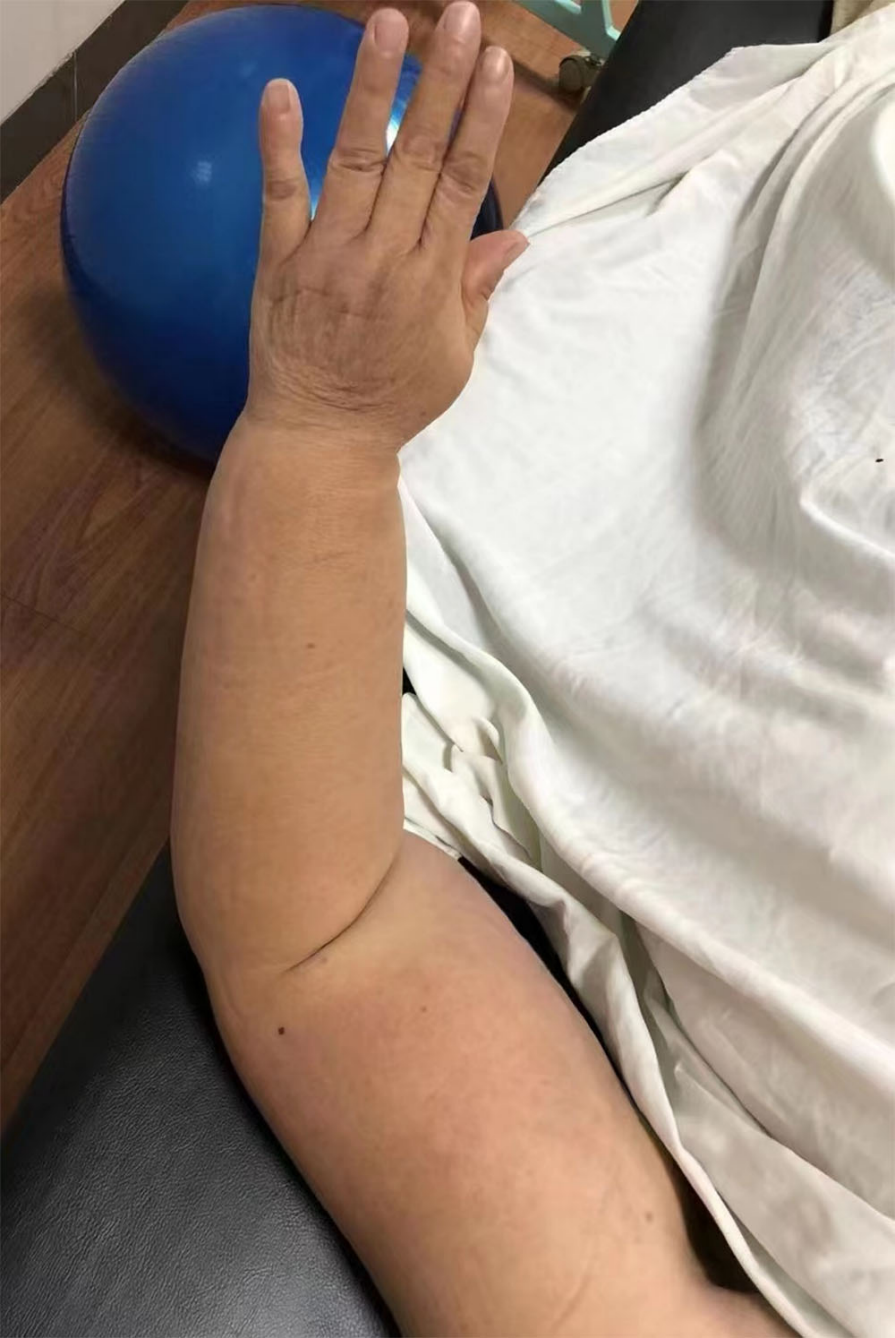
Left Upper Limb Lymphedema After Treatment.

**Figure 5:**
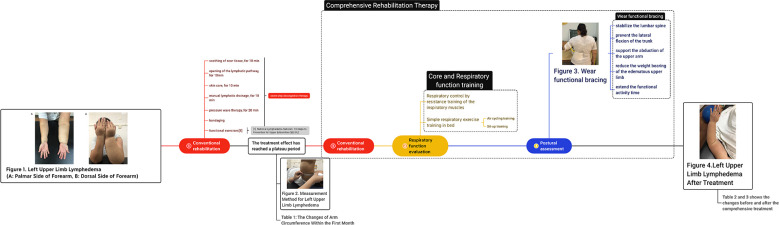
The flow chart of treatment.

During the post-admission examination, it was noted that the left upper limb and left hand were significantly swollen in comparison with the contralateral side. However, there was no elevation in the skin temperature and palpation revealed that the skin was rigid, tensed, and had reduced elasticity. The left upper extremity displayed hypoesthesia and was found to have positive pitting and Stemmer indications. (**Figure 1**) Ultrasound examination of the axilla indicated that neither the deep nor superficial veins of the left upper limb were thrombosed. **Figure 2** illustrates the specific measurement method used. Initially, the patient underwent a conventional rehabilitation program with seven-step decongestion therapy (14) for a duration of one month, and **Table 1** showed an improvement in patient’s left upper lymphedema. But the rehabilitation progress was sluggish and unsatisfactory. To enhance the progress of rehabilitation and identify any additional factors that might be hindering the recovery, a comprehensive rehabilitation therapy was adopted, including the conventional seven-step decongestion therapy, the posture and respiratory assessment combined with their corresponding training and bracing wearing (details can be seen in the Supplement). According to the assessment, the patient exhibited abnormal posture, weakened strength in the respiratory and core muscles, and was incapable of sustaining a functional position of the left upper limb for prolonged periods. The substantial edema in the patient’s left upper limb caused an imbalance in the strength between the left and right sides of the body, leading to an unnatural posture. Based on the reassessment findings, there was implementation of core and respiratory function training as well as utilization of a functional brace (i.e., upper arm-waist fixing brace) (**Figure 3**). A brace was designed and utilized to stabilize the lumbar spine, prevent lateral trunk flexion, and support upper arm abduction during upper-limb activities. The brace also reduced weight bearing of the edematous upper limb and allowed for extended functional activity time. A low-temp. thermoplastic sheet brace was selected due to its good shaping properties, and lightweight, breathability, high strength, and waterproof nature. Appropriate sheets were chosen and limb part sizes were measured. Low-temp. thermoplastic sheets of 3 different specifications and models were selected based on hardness and breathability needed for different fixations. Sheets were put in 65-70°C water and heated for 1-3 min. Softened materials were removed, dried, and shaped. Hook-and-loop fasteners and screws were used to fix, with padding for protection. Elastic sleeves should be worn on affected limb before wearing. To wear, first fix trunk part and Velcro, then upper arm part and Velcro, and adjust to prevent skin compression. Release Velcro every 2 hrs to relieve local pressure. **Tables 2**, **3** shows the changes before and after the comprehensive rehabilitation therapy. **Figure 4** shows the edema of the patient’s left upper limb after treatment. The treatment flow chart outlined the whole intervention process (**Figure 5**).

**Table 1:** The changes of arm circumference within the first month.

Days	Measured from the ulnar styloid process to the shoulder with 10 cm intervals				
	0	10 cm	20 cm	30 cm	40 cm
**1**	22	33	38	38	45
**2**	21.5	32	37.5	37	44
**3**	21	32	38	36.5	43.5
**4**	21	32	38	36	43
**5**	20.5	31.7	37.8	37	43.7
**6**	20.5	31.7	37	37	43
**7**	21.9	32.5	38	37	42.5
**8**	20.4	32.3	37.5	37	43
**9**	20	32.3	37.6	37	43
**10**	20.5	32.3	37.5	37.2	43.5
**11**	20.5	32	37	36.8	43.5
**12**	20	32	37	36.9	43.5
**13**	20	32	37	36.8	43.5
**14**	20	32	36.5	36.5	43.5
**15**	19.5	32	36.5	36.6	43.5
**16**	20	31.9	36.6	36.4	43.5
**17**	19.5	31.9	36.4	36.5	43.5
**18**	19.5	31.8	36.2	36.3	43.5
**19**	20	31.9	36	36.3	43.5
**20**	19.5	31.8	36.1	36.2	43.5
**21**	19.5	31.8	35.8	36.2	43.5
**22**	19.5	31.8	35.7	36.3	43.5
**23**	19.5	31.8	35.5	36.2	43.5
**24**	19	31.7	35.4	36	43.5
**25**	19	31.7	35	36	43.5
**26**	19	31.7	35	36	43.5
**27**	19	31.7	35	36	43.5
**28**	19	31.7	35	36	43.5
**29**	19	31.7	35	36	43.5
**30**	19	31.7	35	36	43.5

**Table 2:** The changes of arm circumference.

Comparisons	Measured from the ulnar styloid process to the shoulder with 10 cm intervals				
	0	10 cm	20 cm	30 cm	40 cm
The left arm circumference (cm)					
** Before the comprehensive treatment**	22	33	38	38	45
** After the comprehensive treatment**	18	27	33.8	33	39
The difference (D) between the two arms about the circumference (cm)					
** Before the comprehensive treatment**	14	11.5	9	14	9
** After the comprehensive treatment**	10	5.5	4.8	9	3

**Table 3:** Changes before and after the comprehensive treatment.

	Before the comprehensive treatment	After the comprehensive treatment
**Range of forward flexion**	0-150°	0-160°
**Range of shoulder abduction**	0-140°	0-155°
**Range of elbow flextion**	0-110°	0-120°
**Range of wrist dorsiflex**	0-30°	0-30°
**Muscle strength Grade of the left upper arm**	The deltoid and extensor carpi muscles are Grade 4, with a grip strength of 10 kg; another muscles are Grade 5.	All the left upper arm muscles are Grade 5.
**The Barthel Index of Activities of Daily Living (ADL), (points)**	95	100
**The Functional Assessment of Cancer Therapy: Breast (FACT-B), (points)**	53	79
Physical well-being	10	13
Social/family well-being	15	20
Emotional well-being	3	15
Functional well-being	15	18
Additional concerns	10	13
**Kessler Psychological Distress Scale, (points)**	24	17

The Ethics Committee of the Affiliated Haikou Hospital of Xiangya Medical College has approved this case report, and the informed consent of the patient has been obtained.

## Discussion

The prevailing view in most studies is that lymphatic obstruction is the underlying cause of upper-limb lymphedema following breast cancer surgery, although the exact mechanism remains unclear (7–10). Treatments such as surgery, radiotherapy, chemotherapy, and others can obstruct or disrupt the lymphatic return pathway in the upper limb. This can result in the retention of lymphatic fluid containing protein in the interstitial spaces, which in turn increases colloid osmotic pressure, and reduces the difference between inside and outside the blood vessels. Consequently, a large amount of fluid from capillaries can enter these spaces. Proteins present in the lymphatic fluid can stimulate the multiplication and release of collagen by fibroblasts. This can lead to fibrosis of subcutaneous tissue and inhibit lymphatic return. As a result of lymphatic vessel dilation and edema, the walls of the vessels thicken and harden and fibrinogen emboli may appear in the lumen. This obstructs lymphatic return and sets off a vicious cycle (1).

According to Stanton et al. (15) long-term work overload can lead to lymphatic pump failure and decompensation, which can eventually cause lymphedema. Bates (16) proposed the concept of interstitial space pressure dysregulation as the potential mechanics for this process. In addition, postoperative radiation can lead to fibrosis and further damage to lymphatic vessels regions such as the proximal limb, axillary, thoracic, and cervical regions where lymphatic fluid stagnation already exist. This can negatively impact, lymphatic return and worsen limb edema.

The standard treatment for lymphedema is complex decongestion therapy (CDT) (17). This therapy typically includes skin care, lymphatic drainage, elastic bandage compression, and functional exercise of the affected limb as part of the international CDT protocol (18). Seven-step CDT is a technique that aims to open the lymphatic route, soothe scar tissue, and utilize CDT-based pressure wave therapy. Shockwave therapy has shown potential benefits on BCRL according to previous research. One possible mechanism is that stretching the skin creates tension on the anchoring filaments, which pulls the Lymphatic Endothelial Cell (LEC) and allows junctions between LEC to open. This leads to fluid accumulation entering the lymphatic lumen and being collected. Additionally, it has been found to reduce skin fibrosis and impact the molecular aspects of lymphangiogenesis. However, there are limitations to its use, including mode (focused or radial), treatment area, treatment frequency, and dosage. When used in combination with CDT, it can significantly improve the volume of lymphedema, skin thickness, and shoulder ROM compared to CDT used alone. However, the current evidence for these benefits is of low methodological quality (19). Previous studies have demonstrated that seven-step CDT following breast cancer surgery can be effective in improving upper-limb lymphedema (14). Liposuction is also a potential treatment option for reducing lymphedema volume, but in this case, the patient declined this treatment option.

In the present study, one month after undergoing seven-step decongestion therapy, the patient’s left upper-limb lymphedema was alleviated. However, further reduction in the lymphedema was not observed after reaching a certain extent, possibly due to subcutaneous tissue fibrosis from prolonged lymphedema after breast cancer surgery (1). It may also have been caused by CDT limitations (1, 18, 19). Thereafter, through the combination of respiratory and core muscle training and the correction of abnormal posture, the patient’s left upper-limb lymphedema was significantly reduced.

Lymphatic fluid circulation relies on various factors such as lymphatic vessel pumping, arterial pulsation, muscle contraction, and thoracic negative pressure. The radical mastectomy of breast cancer destroyed lymph nodes, lymphatic pumping, and surrounding muscle tissue, reducing lymphatic return. In the present study, the patient’s strength, endurance, and coordination of respiratory muscles were improved, abnormal posture was adjusted, thoracic mobility was improved, thoracic breathing ability was strengthened, and thoracic negative pressure was increased, which boosted lymphatic return (20). Additionally, active rehabilitation has been shown to promote lymphatic vessel regeneration and restore vessel continuity by establishing extensive connections between normal tissues containing lymph nodes and those with lymphatic obstruction, thereby draining excessive lymphatic fluid from edematous areas (11).

We studied the effects of comprehensive rehabilitation therapy on lymphedema following breast cancer surgery. The patient in this case had persistent lymphedema and subcutaneous tissue fibrosis as a result of the surgery, and the efficacy of seven-step CDT alone was limited in the sequelae stage of breast cancer surgery. Moreover, by conducting a targeted assessment of the patient’s dysfunction, we were able to identify the underlying cause and provide specific training. Additionally, a custom auxiliary brace was used to correct the malfunction.

## Conclusions

Seven-step decongestion therapy is a useful treatment option for managing upper-limb lymphedema in the aftermath of breast cancer surgery. However, this therapy may have limitations for patients with chronic and prolonged conditions. To address these challenges, a comprehensive rehabilitation approach can be employed, which combines seven-step decongestion therapy with core and respiratory function training, and functional brace wearing. This combined approach has been shown to significantly improve lymphedema symptoms, enhance limb function, and ultimately lead to an improved quality of life for patients with long-standing lymphedema.

## Data availability statement

The original contributions presented in the study are included in the article/supplementary material. Further inquiries can be directed to the corresponding author.

## Ethics statement

The studies involving human participants were reviewed and approved by Ethics Committee of Affiliated Haikou Hospital of Xiangya Medical College. The patients/participants provided their written informed consent to participate in this study.

## Author contributions

Conception and design of the research: Y-FS, JQ-S. Acquisition of data: S-SW Analysis and interpretation of the data: X-FL, H-XW. Statistical analysis: L-QT. Obtaining financing: Z-HS. Writing of the manuscript: Y-FS, L-QT. Critical revision of the manuscript for intellectual content: Z-HS, B-HT. All authors contributed to the article and approved the submitted version.
