# Supplementary material for: Revitalizing quality of life: a case report on the beneficial impact of comprehensive rehabilitation therapy in treating upper-limb lymphedema following breast cancer surgery
Source: Front Oncol. 2023 Jun 16;13:1046003. doi: 10.3389/fonc.2023.1046003 (PMC10313100; doi:10.3389/fonc.2023.1046003)
Supplement: Supplementary file 1 [file DataSheet_1.docx]

**Details of Posture assessment**

The significant edema of the patient's left upper limb caused uneven strength on the left and right sides of the body, and the effort to maintain body balance further caused abnormal body posture. The patient showed the following characteristics in the supine, sitting, and standing positions and when walking: ①Supine position: The head was tilted to the left and rotated to the right rear side. The respiration was predominantly thoracic, the right pelvis was raised, both lower limbs were considerably externally rotated, and there was a functional leg length disparity (the right leg appeared to be shorter).②Sitting position: Viewed from the front, the head was tilted to the left and rotated to the right rear side. The horizontal line of the left acromion was slightly higher than that of the right acromion, and the trunk had slight lateral flexion to the right. Viewed from behind, the left shoulder girdle was rotated downward, and the medial border of the scapula protruded backward. Viewed from the side, the patient assumed a hunched-over head-forward posture. ③Standing position: Viewed from the front, the head was tilted to the left and rotated to the right rear side. The horizontal line of the left acromion was slightly higher than that of the right acromion. The trunk had slight lateral flexion to the right, and the feet were standing with toes pointing outward. Viewed from behind, the left shoulder girdle was rotated downward, and the medial border of the scapula protruded backward. Viewed from the side, the patient assumed a hunched-over head-forward posture, and the pelvis was tilted anteriorly. ④ Gait analysis: The patient had a Trendelenburg gait when walking.

**Details and Results of Respiratory assessment**

The approach utilized the PowerBreathe K5 deep-breathing trainer and was as follows: After launching the software and entering the patient's details, the test was initiated. The respiratory valve was fitted with a filter, and the therapist told the patient to hold the filter in their mouth and take 30 rapid inhalations and gradual exhalations. The evaluation was ended after the patient complained of substantial weariness after 20 breaths.

Results of respiratory assessment showed that ① the patient’s best result for muscle strength index was 25.30 cmH_2_O. Generally, the respiratory function is considered normal when the muscle strength index reaches more than 80% of the normal predicted value, although the index of the patient in this study reached only 29.08%. ② The patient’s best peak inspiratory flow rate was 1.31 L/s, which reflects the ability of the inspiratory muscles to contract rapidly and overcome resistance.

**Core and respiratory function training**

*Respiratory control by resistance training of the inspiratory muscles.* A weighted object (such as a dumbbell or sandbag) was placed on top of the patient's belly after they assumed a supine position with their upper limbs stretched and relaxed on the bed surface and their lower limbs contracted. The therapist requested the following abdominal respiration training from the patient: By breathing through the nose, the abdomen is progressively elevated and held for three seconds following exhalation. After exhaling via the mouth, the abdomen was gradually relaxed. One set consisted of ten breaths, and three sets were performed every day for one month. *Bed Simple breathing exercise training includes aerial cycling training and sit-up training.* Three minutes of training constituted one set, and three sets were performed daily for one month.
